# Mechanisms of Resistance to Antibody-Drug Conjugates

**DOI:** 10.3390/ijms24119674

**Published:** 2023-06-02

**Authors:** Rita Khoury, Khalil Saleh, Nadine Khalife, Mohamad Saleh, Claude Chahine, Rebecca Ibrahim, Axel Lecesne

**Affiliations:** 1 International Department, Gustave Roussy Cancer Campus, 94800 Villejuif, France; rita.khoury011@gmail.com (R.K.); claude.chahine@gustaveroussy.fr (C.C.); rebecca.ibrahim@gustaveroussy.fr (R.I.); axel.lecesne@gustaveroussy.fr (A.L.); 2 Department of Head and Neck Oncology, Gustave Roussy Cancer Campus, 94800 Villejuif, France; khalife.nadine@live.com; 3 Department of Hematology and Oncology, Lebanese American University Medical Center–Rizk Hopsital, Beirut 1100, Lebanon; mohammad.saleh92@outlook.com

**Keywords:** antibody-drug conjugate, trastuzumab emtasine, trastuzumab deruxtecan, enfortumab vedotin, sacituzumab govitecan, monoclonal antibodies, payload, linker

## Abstract

The treatment of cancer patients has dramatically changed over the past decades with the advent of monoclonal antibodies, immune-checkpoint inhibitors, bispecific antibodies, and innovative T-cell therapy. Antibody-drug conjugates (ADCs) have also revolutionized the treatment of cancer. Several ADCs have already been approved in hematology and clinical oncology, such as trastuzumab emtansine (T-DM1), trastuzumab deruxtecan (T-DXd), and sacituzumab govitecan (SG) for the treatment of metastatic breast cancer, and enfortumab vedotin (EV) for the treatment of urothelial carcinoma. The efficacy of ADCs is limited by the emergence of resistance due to different mechanisms, such as antigen-related resistance, failure of internalization, impaired lysosomal function, and other mechanisms. In this review, we summarize the clinical data that contributed to the approval of T-DM1, T-DXd, SG, and EV. We also discuss the different mechanisms of resistance to ADCs, as well as the ways to overcome this resistance, such as bispecific ADCs and the combination of ADCs with immune-checkpoint inhibitors or tyrosine-kinase inhibitors.

## 1. Introduction

The treatment landscape of cancer patients has drastically changed over the last three decades. Initially, chemotherapy was used, from the beginning of the 20th century, with substantial clinical benefits. However, the use of chemotherapeutic drugs involves a constant trade-off between the potential benefits and the systemic toxicity of these non-specific agents [1]. The advent of monoclonal antibodies (mAbs) and targeted therapies, such as rituximab and trastuzumab, revolutionized the therapeutic arsenal in clinical oncology [2,3]. More recently, immune-checkpoint inhibitors (ICIs) and bispecific antibodies were approved for the management of cancer patients. Antibody-drug conjugates (ADCs), initially described by Ehrlich, are another promising therapeutic option that deliver toxic drugs directly into tumor cells, offering a highly effective, yet safe, effect by selectively binding to tumor cells through directed antibodies, which provide exceptional affinity and specificity for a specific epitope on the target antigen [4,5,6].

The idea of ADCs was first proposed in the 1970s, but it was not until the 1990s that the first ADCs were developed and tested in clinical trials. The development of ADCs was driven by the desire to create more effective cancer treatments that could target cancer cells with greater precision while sparing healthy tissues [4]. The use of ADCs in clinical trials with animal models dates back to 1980s when trials were conducted using mouse immunoglobulin G (IgG) [7]. In fact, the choice of IgG isotype for use in ADCs depends on several factors, including the target antigen, the mechanism of action of the ADC, and the intended clinical application. However, IgG1-based mAbs are more commonly used because of their easier production and stronger immunogenic properties compared to the other igG2 and igG4 subtypes [8].

This revolutionary era began with the promising data from the use of BR96-doxorubicin immunoconjugates for the treatment of human carcinomas in animal models. The study found that the BR96-doxorubicin immunoconjugates bound efficiently to Lewis Y, an antigen that is abundantly expressed in cancer cells; thus, they were effective in treating xenografted human carcinomas in mouse models, with some tumors actually achieving complete response. The study also found that the treatment was well tolerated by the animals, with no significant side effects observed [9,10].

Several ADCs are currently approved and used in clinical practice. This review focuses on the structure of ADCs, the mechanisms of action of four selected approved ADCs in solid tumors (ado-trastuzumab emtansine (T-DM1), trastuzumab deruxtecan (T-DXd), sacituzumab govitecan (SG), and enfortumab vedotin (EV)) and the mechanisms of resistance to these agents, which are well known in comparison with other recently approved ADCs.

## 2. ADC Structure

**Figure 1:**
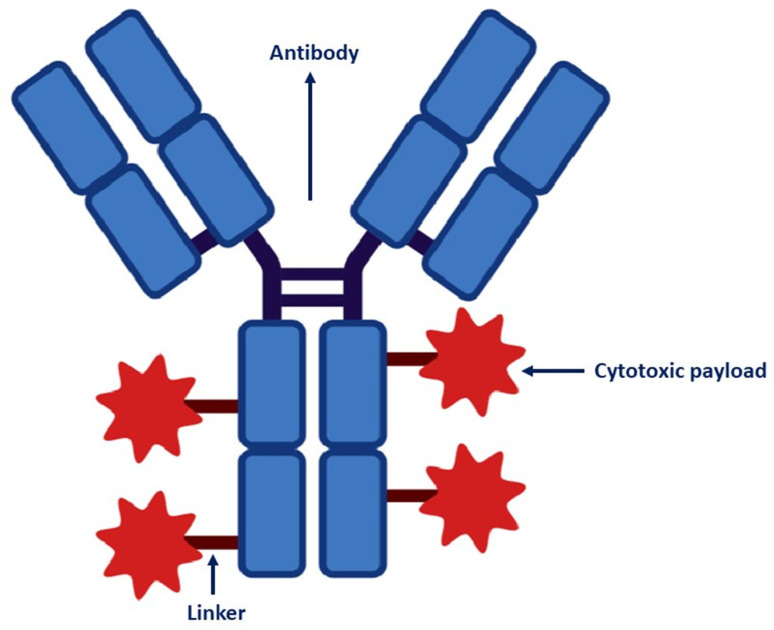
Structure of ADC.

The components of ADCs are humanized mAbs that bind to a tumor-specific or associated antigen and a cytotoxic drug (payload) with anti-tumor activity conjugated through a molecular cleavable or non-cleavable linker [11]. Figure 1 illustrates the structure of an ADC.

### 2.1. Target-Antigen Selection and Antibody

The guiding signal for ADCs to locate tumor cells is the target antigen expressed on the surfaces of tumor cells [12]. The targeted antigen must be expressed exclusively or mostly in tumor cells, with minimal or negligible expression in normal tissues, in order to minimize off-target damage [13]. Antigens that are only expressed on the surfaces of tumor cells make excellent targets for ADCs. With many particular ADCs already approved and others in development, lineage-specific antigens expressed by hematological malignancies have been thoroughly investigated as ideal candidates [11]. However, this concept does not apply to solid tumors, in which the antigens expressed are primarily “tumor-associated” rather than “tumor-specific,” meaning that they are expressed on both tumor cells and normal cells [8].

The antibody consists of two antigen-binding fragments (mainly known as Fabs) and one constant fragment (Fc). The antigen recognition is mediated by Fabs, and the interaction of the antibody with effector immune cells is mediated by Fc [6]. The integrated antibody should have a high level of specificity for the tumor’s target antigen. An optimal antibody molecule should have high binding affinity with the target antigen, as well as effective internalization and minimal immunogenicity, and it should feature a long plasma half-life [14]. Due to its advantage of possessing much lower immunogenicity than mouse-derived antibodies, developed antibodies are now fully humanized. Since IgG is the primary component of immunoglobulin in serum, the antibodies now employed in ADC medications are primarily IgG antibodies, which encompass four subtypes, namely IgG1, IgG2, IgG3, and IgG4 [12]. The IgG1 subtype is the most commonly used for antibody therapeutics [15]. The immune-effector functions mediated by the antibody are the activation of the complement complexes, the activation of immune cells, the induction of antibody-dependent cell-mediated cytotoxicity (ADCC) and complement-dependent cytotoxicity (CDC), and the activation of antibody-dependent cell-mediated phagocytosis (ADCP).

### 2.2. Cytotoxic Payloads

The cytotoxic payload is the major effector component of the ADC, and it is highly potent. Currently, the primary cytotoxic payloads for ADCs include potent tubulin inhibitors (auristatin analogs and maytansine analogs), DNA-damaging compounds (duocarmazine, calicheamicins, and pyrrolobenzodiazepines), the RNA polymerase II inhibitor (amanitin), and topoisomerase I inhibitors (deruxtecan, govitecan) [1]. It was demonstrated that the molecular and physicochemical characteristics of the payload are important for the efficacy of the ADC, in addition to its potency.

### 2.3. Linkers

The linkers connect the antibodies to the cytotoxic payloads and contribute to the stabilization of the ADCs [16]. These linkers can be non-cleavable, in which case they require more processing to release the payload, or cleavable, in which case they are related to tumor-specific factors, such lysosomal enzymes or changes in pH (low pH). It was demonstrated that cleavable linkers have the advantage of releasing the cytotoxic payload more efficiently. However, non-cleavable linkers may release their chemotherapy payloads with more specificity, but the antibody needs to be completely degraded in the lysosomes that might affect the payload. Non-cleavable linkers are associated with higher plasma stability and relatively longer half-lives [17,18,19].

The ADCs can be categorized by an important indicator, the drug-to-antibody ratio (DAR). The DAR represents the number of cytotoxic moieties attached to each antibody. The ADCs with the highest DAR might be more potent, but they have faster drug clearance and, potentially, increased toxicity. However, ADCs with lower DAR might have lower activities but higher therapeutic indices [20,21].

## 3. Selected ADCs in Solid Tumors: Mechanism of Action

As previously mentioned, we focus in this review on four ADCs: T-DM1, T-DXd (formerly known DS-8201), SG, and EV. Table 1 summarizes the key trials leading to the approval of these agents for the treatment of cancer patients.

### 3.1. Trastuzumab Emtansine

The T-DM1 was the first ADC approved for metastatic breast cancer (MBC) based on the results of the EMILIA trial [31]. It works through a variety of methods, including the selective delivery of DM1 (a microtubule-inhibitory-agent derivative of maytansine) to HER2-positive tumor cells, the trastuzumab-mediated suppression of HER2 signaling, the prevention of the shedding of the HER2 extracellular domain, and the induction of ADCC [32,33,34].

First, T-DM1 selectively binds to subdomain IV of the HER-2-receptor-positive cells. After binding, the ADC–receptor complex is internalized into endosomes by endocytosis [34]. Endocytic vesicles either develop and join the lysosome or are recycled to return the ADC–receptor complex to the plasma membrane [35]. The antibody component of T-DM1 undergoes lysosomal breakdown, releasing lysine-MCC-DM1 [36]. After its release from lysosomes, MCC-DM1 effectively attaches to tubulin to stop microtubule polymerization, resulting in cell-cycle arrest [37].

It was shown that T-DM1 retains the mechanisms of action of trastuzumab, including the suppression of HER2-ectodomain shedding, the blockage of HER2-signaling pathways, and the stimulation of innate and adaptive anti-tumor immunity [32]. It works by reducing the ligand-independent activation of the downstream phosphatidylinositol 3-kinase (PI3K)–AKT–mammalian target of rapamycin (mTOR) and RAS–mitogen-activated protein kinase (MAPK)-signaling pathways [38]. Trastuzumab’s Fc domain is recognized by homologous Fc receptors on natural killer cells, resulting in ADCC and the destruction or phagocytosis of HER2-positive tumor cells that are tagged by trastuzumab [39]. Trastuzumab was linked to a number of additional, less conclusively shown modes of action, including DNA-repair inhibition, the increased accumulation of the cyclin-dependent kinase inhibitor p27Kip1, and the suppression of angiogenesis [40].

### 3.2. Trastuzumab Deruxtecan

T-DXd is a next-generation ADC that consists of a humanized anti-HER2 antibody linked to deruxtecan, a potent topoisomerase I inhibitor (exatecan derivative, DXd), through a tetrapeptide cleavable linker. It has been approved for patients with MBC [41]. Trastuzumab deruxtecan is characterized by several innovative features, such as its effective targeting of tumors with low HER2, as well as its powerful cytotoxic payload, with a drug-to-antibody ratio of 8:1, which allows it to target neighboring cells [42,43]. After the positive results and durable activity seen in HER2-positive patients in phase I and II trials, T-Dxd not only dethroned standard chemotherapy in the second-line setting and resulted in improvements in progression-free survival (PFS) and overall survival (OS) in patients already treated with T-DM1, but also demonstrated activity in patients with low-Her2 tumors [22,44,45]. Furthermore, T-DXd has also been approved for the treatment of patients with metastatic HER2-positive gastric or gastroesophageal junction adenocarcinoma who received a prior trastuzumab-based regimen and those with previously treated HER2-mutant metastatic non-small-cell lung cancer (NSCLC), based on the results of the DESTINY-Gastric 01 and DESTINY-Lung01 phase II trials [24,25].

### 3.3. Sacituzumab Govitecan

An active metabolite of irinotecan, SN-38 is chemically linked to the humanized monoclonal RS7 IgG1 Trop-2 antibody that makes up SG [46].

It was the first ADC approved for triple-negative breast cancer (TNBC) based on the results of the IMMU-132-01 and ASCENT trials [26,47]. It has also been approved for the treatment of metastatic urothelial carcinoma (mUC) after progression on ICIs and, more recently, for the treatment of HR+/HER2- MBC after progression on endocrine therapy and at least two lines of chemotherapy [27,28]. It is internalized after binding to Trop-2 on cancer cells, enabling SN-38 to be delivered intracellularly [48]. Furthermore, SN-38 is around 1000 times more potent in causing DNA breaks [49]. Sacituzumab Govitecan is characterized by a relatively high DAR and a cleavable CL2A linker [50]. The CL2A linker, which has intermediate stability, is used to enable SN-38 release in sacituzumab-bound cells, as well as in the tumor microenvironment (TME). The anti-tumor efficacy of this drug is probably increased by the therapeutic SN-38 concentrations that are produced in the cells with conjugate binding and the TME [51].

### 3.4. Enfortumab Vedotin

Enfortumab Vedotin is an ADC made of a human IgG1 anti-Nectin-4 antibody coupled to monomethyl auristatin E (MMAE), a molecule that disrupts microtubules [52]. It is approved as a monotherapy for the treatment of patients with mUC after progression on ICIs [29]. On April 2023, EV was approved in combination with pembrolizumab for the treatment of patients with mUC ineligible for cisplatin-containing chemotherapy, based on the results of the EV-103 trial [30].

Nectin 4 is an optimal therapeutic target as it is overexpressed by multiple malignancies, but is especially common in urothelial cancers and breast cancer [53]. When EV attaches to nectin-4 and internalizes, the microtubule-disrupting substance MMAE is released intracellularly, which causes the tumor cell to undergo apoptosis [54]. The antitumor effects of EV can also be mediated by additional mechanisms, such as the inhibition of signal transduction from direct binding, ADCC, and CDC [55].

## 4. Mechanisms of Resistance to ADC

**Figure 2:**
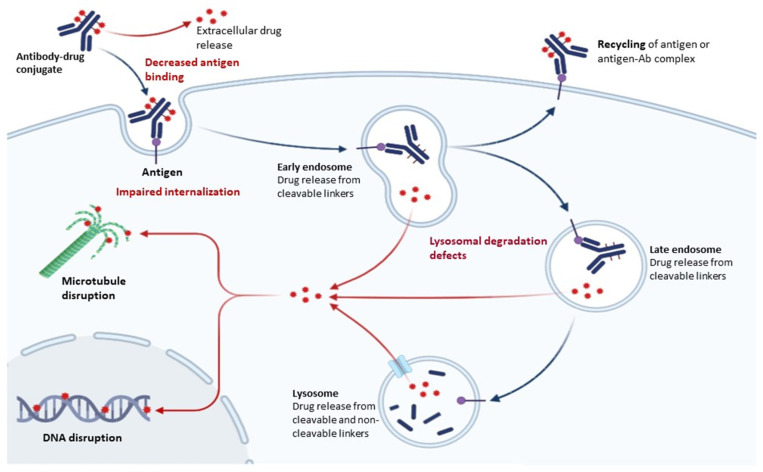
Mechanisms of resistance and of overcoming of resistance.

As the structures of ADCs are complex and their mechanism of action consists of multiple steps, resistance can occur at any level, beginning with antigen expression and recognition, through internalization and degradation to cytotoxic drug release and apoptotic regulation. Table 2 summarizes the mechanisms of resistance and those through which resistance can be overcome. Figure 2 illustrates these mechanisms.

### 4.1. Antigen-Related Resistance

Since ADCs work by targeting specific antigens, one of the proposed mechanisms of resistance is related to the recognition of these antigens by mAbs. Loganzo et al. conducted a study in which they exposed breast cancer cells to trastuzumab–maytansinoid (TM–ADC), an ADC that is structurally similar to TDM-1. They used two different cell lines, MDA MB-361-DYT2 cells, which are parental cells, and JIMT1 cells, which are cancer cells resistant to first-line trastuzumab. Interestingly, different mechanisms of resistance were observed after the exposure to TM–ADC. The JIMT1–TM-cell models developed resistance to TM–ADC while remaining sensitive to other chemotherapeutic options, including tubulin inhibitors, supporting the theory that the reduced HER2 protein levels following months of treatment might have led to refractory cells and, eventually, drug resistance [56].

Furthermore, tumor heterogeneity in antigen expression might in fact affect the efficacy of ADCs. This phenomenon was described in the KRISTINE trial, a phase II study of T-DM1 plus pertuzumab in the neoadjuvant setting. The heterogeneity of HER2 was defined as the detection of a HER2-negative area by FISH in 10% of the cases or ERBB2 amplification in more than 5% but less than 50% of tumor cells. The primary endpoint was not met in any of the ten patients exhibiting tumor heterogeneity, with a pathologic complete response (pCR) of 0% [57]. The results of the trial showed that the patients who had a high level of heterogeneity in HER2 expression before their treatment had worse PFS and OS compared to those with low heterogeneity [57]. This was also assessed in the ZEPHIR trial, in which patients who expressed high tumor heterogeneity on a HER2-PET CT scan experienced shorter times to treatment failure [58].

Another potential resistance mechanism, which has not yet been clinically proven in ADC, is the buildup of truncated forms of the antigen ectodomain. In fact, this theory was previously demonstrated with trastuzumab, when tumors expressing full-length receptors showed high responses to trastuzumab, whereas truncated P95HER2 exhibited resistance [59]. Furthermore, the masking and isolation of the HER2 antigen by extracellular matrices such as MUC4 led to trastuzumab refractoriness in JIMT-1, presenting another probable mechanism of resistance [60].

Lastly, sensitivity to ADCs can be modulated by the presence of ligands such as Heregulin, also known as neuregulin or NRG, a protein that belongs to the epidermal growth factor (EGF) family of proteins that impairs that efficacy of T-DM1 by encouraging the heterodimerization of HER2 with HER3 and HER4 [61].

Similarly, resistance to SG was assessed by Coates et al. [62]. The authors reported a case study of a patient with metastatic TNBC who initially responded to SG therapy but eventually developed acquired resistance. The study found that the patient’s tumor acquired multiple genomic alterations that affected both the antigen target (Trop-2) and the drug payload (SN-38). These alterations included mutations, copy-number changes, and structural variations, which resulted in reduced Trop-2 expression and increased drug efflux, leading to reduced drug exposure and resistance to SG therapy. The findings suggest that acquired resistance to SG in TNBC is mediated by parallel genomic alterations of both the antigen and the payload targets [62].

On a similar note, Nectin-4 expression and its association with EV was evaluated by Klumper et al. Their article showed that membranous Nectin-4 expression frequently decreases during the metastatic spread of UC, suggesting that NECTIN-4 may be downregulated during the progression of the disease. Furthermore, the article demonstrated that Nectin-4 downregulation is associated with resistance to EV therapy as absent or weak expressions were correlated with shortened PFS. These findings suggest that monitoring Nectin-4 expression in UC patients may be useful for predicting responses to EV therapy and identifying patients who may benefit from alternative treatments [63].

### 4.2. Payload-Related Resistance

In addition to antigen resistance, tumor cells might develop payload resistance. This theory was observed initially with NHL tumors, in which the replacement of an auristatin-based payload with an anthracycline-based payload resulted in a better response to ADC [64]. Similarly, another example was illustrated by Takegawa et al., in their work with T-DXd in T-DM1-resistant cells. In fact, in that study, the T-DM1-resistant cells retained normal HER2 overexpression but showed an upregulated expression of the ABC transporters ABCC2 and ABCG2. Moreover, the T-DM1 efficacy was restored by inhibiting these transporters, shedding light on the mechanism of resistance that is caused by amplified DM1 efflux [65]. This theory was further supported by the ongoing phase I/II studies for SKB-264, a novel TROP-2-targeted ADC, in which the topoisomerase-1 inhibitor’s payload was replaced with a belotecan derivative [66]. Payload diversification is a promising approach to ADC therapeutic effects. On a similar note, in addition to the type of payload, location of the conjugations location and the average DAR are significant. Payloads are conjugated to antibodies through either cysteine (Cys) or lysine (Lys) residues [67]. Hamblett et al. reported an optimal average of 2–4 DAR in order to achieve the best balance in cysteine-linked ADCs, whereas lysine conjugates required 3–4 DAR [68]. In one case study, it was found that lysine-conjugated ADCs led to better results than cysteine-conjugated ADCs. These ADCs had the same antibody and the same cytotoxic payload [69]. However, another comparison was made using DGN549 as a payload. In this case, site-specific cysteine-based conjugation showed improved tolerability and better efficacy. These cases support the argument that conjugation chemistry is best evaluated on each and every antibody, target, and linker payload, as the results might not be consistent [70]. Finally, concerning payloads, DAR plays a major role in ADC efficacy. However, there is no clear number stating the optimal DAR. One study assessed the potency of ADCs with similar cytotoxic agents at the same time as their DAR. The preclinical data suggested that the ADCs with very high DAR (~9–10) had faster clearance than those with lower ADCs, and, therefore, that they had decreased efficacy [20]. However, other factors might also affect these values, such as the site of conjugation and drug loading.

### 4.3. Failure in Internalization and Trafficking Pathways

Once the ADC binds to the target antigen, it is internalized into the cancer cell by endocytosis. There a several routes of endocytosis, including clathrin-mediated endocytosis (CME), which is the most common route adopted by ADCs, caveolae-mediated endocytosis, and clathrin-caveolin-independent endocytosis [71].

Each route of endocytosis is regulated by a distinct set of proteins, such as the adaptor protein (AP2), dynamin, epsin, and phosphatidylinositol biphosphate (PIP2), and each plays a specific role in various cellular processes.

In a study conducted by Sung et al., several in vitro models of T-DM1 resistance were generated, despite the use the same approach. Two models exhibited resistance via the aforementioned mechanism (reduced HER2 expression), whereas N87-TM showed normal expressions of transport protein and retained similar levels of HER2 to the parental cells [66]. Moreover, these resistant N87-TM cells were in fact able to internalize trastuzumab-ADCs into caveolin-1 (CAV-1)-coated vesicles. These data suggest that the microenvironment and enzymes play an important role in the antibody catabolism and delivery, favoring the cleavable over the non-cleavable linker payload of TDM-1 [72].

In addition, in HER2-positive breast cancer, Endophilin A2 (encoded by SH3GL1) has been shown to increase HER2 internalization and enhance the sensitivity of breast cancer cells to trastuzumab-based therapy. The knockdown of SH3GL1 in tumor cells led to reduced internalization and evidently suppressed T-DM1-mediated cytotoxicity [73].

### 4.4. Impaired Lysosomal Function

After binding to the target molecule and internalization via receptor-mediated endocytosis, ADCs reach lysosomes, where chemical or enzymatic cleavage induce the release of the cytotoxic agents. However, several causes might lead to impaired function.

Rios-Luci et al. reported data on T-DM1-resistance mechanisms by isolating three different resistant HER2-positive clones. Compared to parental clones, these resistant clones showed similar HER2 expressions, with unaltered internalization and trafficking pathways. Nevertheless, the latter clones displayed higher lysosomal pH levels, with disturbed proteolytic activity and, consequently, accumulated levels of T-DM1 [74]. This poor lysosomal function ultimately leads to impaired T-DM1 processing, limiting its anti-tumor activity. The description of this deficient activity not only helps to explain the different ADC-mechanism pathways, but might also help to overcome them. It is interesting to mention a published report by Trudeau et al., who used UV photoactivation to create acidifying nanoparticles that manipulate intralysosomal pH and eventually restore or augment the anti-tumoral properties of ADCs [75].

A different mechanism of resistance to ADCs is associated with the transport of cytotoxic drugs from the lumens of lysosomes to the cytoplasm. This mechanism is of special interest in non-cleavable linkers, as they may require a special transporter to be delivered into the cytoplasm. Hamblett et al. performed a phenotypic RNA screening, revealing SLC46A3 as a direct transporter of maytansine-based catabolites to the cytoplasm. The silencing of this protein results in catabolite accumulation in the lysosomes and, eventually, drug failure [76].

Moreover, resistance mechanisms to T-DXd were evaluated in the DAISY trial. This was a phase II, multicenter, open-label trial enrolling patients in three groups, HER2+, HER20, and low-HER2, to whom T-DXd is administered at a dose of 5.4 mg/kg every 3 weeks, with the best objective response as the primary endpoint. Upon progression, the tumors were assessed by whole-genome sequencing (WES) in order to detect potential resistance mechanisms [77]. In addition to decreases in HER2 expression, mutations in SLX4 gene might result in resistance cells. The SLX4 gene plays a critical role in DNA-damage repair and regulates three endonucleases [78]. This study demonstrated that tumor cells with depleted SLX4 exhibited T-DXd resistance and, thus, loss-of-function mutations might be further mechanisms of T-DXd resistance [77].

### 4.5. Drug-Efflux Pumps

The overexpression of ATP-binding cassette (ABC) transporters plays a major role in chemotherapy resistance, and the correlation between transporter expression and reduced responses to chemotherapy has been documented for many cancer types [79]. These efflux pumps enhance drug extrusion from cells and might lead to the emergence of tumors with multidrug-resistant (MDR) phenotypes [80]. This same mechanism might directly contribute to ADC resistance, given the fact that many of the payloads are substrates of ABC [81,82]. The most commonly seen phenotype is MDR1-mediated efflux. Maytansinoids are substrates of ABC transporters, such as MDR1; thus, resistance to T-DM1 can be potentially linked to substrate expression [83]. Kovtun et al. reported data on possible ways of bypassing multidrug resistance. They used the hydrophilic linker PEG_4_Mal to conjugate the cytotoxic maytansinoid DM1 to the antibody and compared their action to similar conjugates linked with a nonpolar linker, *N*-succinimidyl-4-(maleimidomethyl) cyclohexane-1-carboxylate (SMCC). The PEG_4_Mal-linked conjugates showed greater activity and superior responses in MDR1-expressing tumors [81].

### 4.6. Role of Cell Cycle

Actively cycling leukemic cells were found to be more sensitive to chemotherapy than resting leukemic cells. Therefore, the theory of cell-cycle effects on tumor response is not new [84].

Another proposed mechanism of resistance to T-DM1 is associated with the level of cyclin B, which is a cell0cycle protein that takes part in the G2–M transition. Sabbaghi et al. designed an experiment in which they induced T-DM1 resistance and compared its cellular and molecular effects on parental and resistant cells. The two groups had similar HER2 expressions, binding, and intracellular uptake. However, the researchers detected cyclin B1 accumulation in sensitive cells as opposed to resistant cells. Furthermore, the silencing of the upregulation of cyclin B1 levels led to the partial sensitization of the resistant cells. This proved that cyclin B1 levels suggest the effectiveness of T-DM1 in apoptosis [85].

### 4.7. Activation of Signaling Pathways

The activation of signaling pathways can be one of the mechanisms through which malignant cells develop resistance to ADCs. One of the signaling pathways that can be activated is the PI3K/AKT/m TOR pathway, which is involved in cell survival, growth, and metabolism. Its activation can potentially result in decreased sensitivity to ADCs, reduced effectiveness of the cytotoxic payload, and prolonged cell survival. This was previously described with trastuzumab in patients with PIK3CA mutations or PTEN deletions [84]. In fact, the knocking down of PTEN led to trastuzumab failure; these findings suggest that PTEN loss or PIK3CA hyperactivation might result in decreased sensitivity to trastuzumab through PI3K/AKT-signaling activation.

However, the results from an exploratory biomarker analysis in an EMILIA trial suggest otherwise. In fact, in this phase III trial, T-DM1 not only prolonged both OS and PFS compared to lapatinib and capecitabine in patients with MBC previously treated with trastuzumab and taxane, but also showed similar results in both PIK3CA-mutated and wild-type tumors [31]. Among the patients who were treated with lapatinib and capecitabine, those who carried the PIK3CA mutation or had decreased PTEN expression had shorter PFS and OS. The patients treated with T-DM1 had similar results, regardless of the aforementioned mutations. These data suggest that T-DM1 might be more effective than other HER2-directed therapies, irrespective of the tumor biomarkers [86].

Furthermore, the activation of the Wnt/β-catenin pathway can also encourage resistance to ADCs. Wu et al. described the role of Wnt3 in trastuzumab resistance. The overexpression of Wnt3 led to the increased expression of β-catenin and increases in growth rates and invasiveness, as well as trastuzumab resistance, in these cells [87]. These remain potential, but not proven, resistance mechanisms to ADCs, as they have not yet been directly linked to T-DM1 resistance.

### 4.8. Apoptotic Dysregulation

Finally, any change in apoptotic modulation can change sensitivity to ADCs. Most of the data are derived from hematological malignancies, with the correlation of BCL-2 and BCL-XL overexpression with gemtuzumab ozogamicin or brentuximab vedotin [88,89,90].

## 5. Overcoming Resistance to ADCs

Despite major advancements in ADC technology, resistance remains a significant challenge. As previously discussed, resistance to ADC can arise through a variety of mechanisms, some of which are not yet fully understood. Therefore, the development of strategies to overcome ADC resistance is a complex and ongoing area of research, whether through novel ADC designs or through combination therapy.

One of the most common mechanisms is the increase in drug extrusion through the overexpression of drug-efflux pumps. One example of overcoming ADC resistance was illustrated in the previously described study by Takegawa et al., who found that T-DXd was able to overcome T-DM1 resistance in HER2-positive gastric cancer cells, and that the novel DNA topoisomerase I inhibitor incorporated into the ADC, with aberrant expression of ABC transporters, may be responsible for this enhanced anti-tumor activity [65]. These findings were recently confirmed in DESTINY-Breast02, a phase III trial evaluating T-DXd versus chemotherapy of choice in patients with HER2+ MBC refractory to T-DM1. This was the first randomized trial to show that one ADC can overcome resistance to another [22].

Another strategy relies on hydrophobic compounds, as they are more efficiently transported than hydrophilic compounds. Therefore, the idea consists of modifications to the linker to make it more hydrophilic, and, thus, decrease its elimination. Sulfo-SPDB-DM4, a highly hindered disulfide hydrophilic linker used in FRα-expressing tumors, and PEG_4_Mal are clear examples of enhanced effectiveness against resistant, MDR1-positive tumors [81,91].

A different, yet very important concern is tumor heterogeneity and the low efficacy of ADCs on tumor cells that exhibit low antigen expressions. Golfier et al. investigated the efficacy of anetumab ravtansine, an ADC targeting the protein mesothelin, in curing tumors with heterogeneous target expressions. The study found that Anetumab ravtansine demonstrated potent anti-tumor activity in vitro and in vivo, selectively targeting and killing mesothelin-expressing tumor cells through a bystander effect, which involves the transfer of the drug to neighboring cells that do not express the target protein [92].

Another study, conducted by Li et al., investigated the effect of the amount of payload release on ADC potency. The authors concluded that with higher rates of payload release, greater potency and a stronger bystander effect were noticed, suggesting a possible helpful strategy for bypassing resistance and enhancing the efficacy of ADCs [93]. This so-called bystander effect depends not only on the charge in the cytotoxic linker, but also on the mechanism of action of the cytotoxic payload and the proximity of neighboring cells, making it quite a complex phenomenon. Further research is needed to fully optimize this method. The bystander effect was one of the reported mechanisms for overcoming resistance to T-DM1 using T-DXd.

Another promising solution to low antigen expression is the use of the novel bispecific or biparatropic mAbs. This has already been demonstrated with HER2. A newly engineered biparatropic ADC exhibited unique characteristics and showed benefits for both T-DM1-resistant and low-HER2-low tumors. This superiority was ensured by targeting two different epitopes of HER2 and, consequently, inducing HER2-receptor clusters, enhancing internalization, and redirecting internal trafficking from recycling to degradation. Furthermore, this ADC was coupled to a different payload, helping bypass the efflux pumps [94].

Andreev et al. explored the potential of bispecific ADCs that bridge HER2 and the prolactin receptor (PRLR) to enhance the efficacy of HER2 ADCs. The PRLR is expressed on the surfaces of some HER2-positive cancer cells and can enhance the proliferation and survival of these cells. The authors found that a bispecific antibody that targets both HER2 and PRLR can increase the binding and internalization of HER2-targeted ADCs in vitro and in vivo, resulting in improved antitumor activity in preclinical models of HER2-positive breast and ovarian cancers. Moreover, an ADC that incorporates both HER2 and PRLR-targeting antibodies showed potent antitumor activity against HER2/PRLR co-expressing tumors in vitro and in vivo. These findings suggest that bispecific antibodies and ADCs that target HER2 and PRLR may offer a promising approach for improving the efficacy of HER2-targeted therapies in HER2-positive cancers that co-express PRLR [95].

In addition, preliminary results were recently presented from a phase I trial investigating zanidatamab zovodotin (ZW49), a bispecific antibody directed against two non-overlapping HER2 epitopes, in solid tumors. This was the first in human trial, and it was found that ZW49 had a manageable safety profile, with a response rate of around 31% and a disease-control rate of around 70%. These are encouraging numbers that could eventually drive further clinical development [27].

A new generation of ADCD, SHR-A1811, comprises a humanized HER2-targeting monoclonal antibody (trastuzumab), a cleavable linker, and a novel topoisomerase I inhibitor payload (SHR9265). It was evaluated in a phase I multicenter first-in-humans trial. A total of 250 heavily pretreated patients with HER2-positive breast cancer, HER2+ gastric/GEJ carcinoma, low-HER2 BC, HER2-expressing/mutated NSCLC, or other HER2-expressing/mutated solid tumors were enrolled. The ORR of the entire cohort was 61.6% (154/250 patients). In the HER2+ BC patients, the ORR was 81.5% (88:108) and in the low-HER2 patients it was 55.8% (43/77). The 6-month PFS rate was 73.9% in all the patients. Interestingly, in the patients with HER2+ BC who previously received T-DM1, the ORR was 82.4% (14/17 patients), and it was 60% (9/15) in the patients whose cases were refractory to HER2-ADC other than T-DM1, including T-DXd [96]. The A166 is another HER2-targeting ADC that showed impressive results in patients with HER2+ MBC. The ORR was around 70% and the mPFS ranged from 9.4 to 12.3 months, according to the received doses [97].

Lastly, another approach for overcoming resistance consists of combination therapies. The addition of ICIs to ADCs might upregulate CD8^+^ effector T cells to tumor sites and improve responses [98].

This was investigated in different trials. Several phase Ib trials with different combinations are currently ongoing. The KATE2 study is the first phase II trial to investigate the combination of atezolizumab and T-DM1 in the treatment of HER2+ metastatic BC. It did not show a clinically meaningful PFS improvement [99]. However, a prespecified analysis showed a possible benefit in a PDL1+ population and, thus, the KATE3 trial is currently ongoing to evaluate this same combination in HER2-positive and PDL1-positive tumors [100].

In fact, combination therapy is not only limited to immunotherapy, as the combination of TKI + TDM1 or T-DXd is also undergoing evaluations in several phase I, II, and III trials for possible synergistic effects.

A different combination strategy relies on the targeting of heregulin or NRG1, a ligand that activates the HER3 receptor and is known to be involved in the development of resistance to anti-HER2 therapy [61]. The efficacy of combining two anti-HER2 agents, trastuzumab emtansine (T-DM1) and pertuzumab, was investigated in the treatment of HER2-positive BC. The researchers found that the combination therapy inhibited the NRG1/HER3-signaling pathway and ultimately resulted in more tumor inhibition and prolonged tumor regression [61]. This idea was further discussed by Schwarz et al., who found that resistant cell lines exhibited increased HER3 and NRG1 expression. Treatment with a PAN-HER induced ERBB-receptor downregulation and, eventually, tumor regression in T-DM1-resistant cell lines. In addition, the use of a HER3-neutralizing antibody in NRG1-expressing cells, which are resistant to T-DM1, resensitized them [101].

## 6. Conclusions and Future Perspectives

Currently, ADCs are an important class of cancer therapeutics, with several FDA-approved ADCs currently available for the treatment of various cancers. Despite all the advancements in cancer therapy, inherent and acquired drug resistance continue to be major obstacles to successful treatment. Multiple mechanisms of resistance to ADCs have been reported, including antigen-related resistance, failure in internalization, impaired lysosomal function, drug-efflux pumps, and alterations of targets. Novel approaches are adopted to overcome these mechanisms. Another potential mechanism of resistance to ADCs is mutation in the target of the payload. However, there are no data to support this theory and no reports regarding mutations in tubulin, RNA polymerase II, or topoisomerase I. New-generation ADCs were efficacious after progression on another ADC. It was reported in DESTINTY-Breast02 that T-DXd was superior to standard chemotherapy in patients with HER2+ MBC refractory to T-DM1. Moreover, SHR-A1811 was associated with high ORR in patients with HER2+ MBC refractory to T-DM1 and T-DXd. The second approach is the combination of ADCs with either ICIs or TKIs. Several ongoing clinical trials are evaluating the combination of T-DM1, T-DXd, SG, and EV with ICIs and TKIs. These are summarized in Table 3, Table 4 and Table 5.

## Figures and Tables

**Table 1:** Key trials of T-DM1, T-DXd, SG, and EV that led to FDA approval.

Trial	Drugs	Phase	No. of pts.	Population	Primary Endpoint	Grade ≥ 3 AEs
EMILIA (NCT008291666)	T-DM1 vs. lapatinib + capecitabine	III	991	HER2+ MBC previously treated with taxanes + T	mPFS: 9.6 vs. 6.4 m (*p* < 0.001) mOS: 30.9 vs. 25.1 m (*p* < 0.001)	41% vs. 57% T-DM1: Thrombocytopenia (13%) Elevated ASAT (4%) Anemia (3%)
DESTINY-Breast02 (NCT03523585) [22]	T-DXd vs. T + capecitabine or lapatinib + capecitabine	III	608	HER2+ MBC previously treated with T-DM1	mPFS: 17.8 vs. 6.9 m (*p* < 0.0001)	53% vs. 44% ILD: 10%
DESTINY-Breast 03 (NCT03529110) [23]	T-DXd vs. T-DM1	III	524	HER2+ MBC previously treated with T and taxane	mPFS: not reached vs. 6.8 m 12-mPFS rate (76 vs. 34%) (*p* < 0.0001)	52% vs. 48% T-DXd: Neutropenia (19%) Thrombocytopenia (7%) ILD (1%)
DESTINY-Gastric01 (NCT03329690) [24]	T-DXd vs. physician of choice chemotherapy	II	187	HER2+ gastric cancer receiving at least 2 previous lines including T	ORR: 51% vs. 14% (*p* < 0.001)	Neutropenia (51%) Anemia (38%) Leucopenia (21%) ILD (2%)
DESTINY-Lung01 (NCT03505710) [25]	T-DXd	II	91	Metastatic HER2-mutant NSCLC refractory to standard treatment	ORR: 55% (mPFS: 8.2 m; mOS: 17.8 m)	Neutropenia (19%) Anemia (10%) Nausea (9%)
ASCENT (NCT02574455) [26]	SG vs. single-agent CT	III	468	metastatic TNBC refractory to two prior lines	mPFS: 5.6 vs. 1.7 (*p* < 0.001)	Neutropenia (51%) Leukopenia (10%) Diarrhea (10%) Anemia (8%)
TROPiCS-02 (NCT03901339) [27]	SG vs. single-agent CT	III	543	HR+/HER2- mBC prior taxane, ET, CDK4/6 inhibitor and 2-4 prior CTs	mPFS: 5.5 vs. 4.0 m (*p* < 0.001)	Neutropenia (51%) Diarrhea (10%)
TROPHY-U-01 (NCT03547973) [28]	SG	II	113	mUC progressed after platinum-base CT and ICIs	ORR: 27%	Neutropenia (35%) Leukopenia (18%) Anemia (14%) Diarrhea (10%) Febrile neutropenia (10%)
EV-301 (NCT03474107) [29]	EV vs. docetaxel or paclitaxel of vinflunine	III	608	mUC progressed after platinum-base CT and ICIs	mOS: 12.9 vs. 9.0 m (*p* = 0.001)	Maculopapular rash (7%) Fatigue (6%) Neutropenia (5%)
EV-103 (NCT04223856) [30]	EV + pembro	Ib/II	45	mUC first-line cisplatin-ineligible pts	ORR 73% including 16% CR	Increased lipase (18%) Maculopapular rash (11%) Fatigue (11%)

T-DM1: trastuzumab emtansine; MBC: metastatic breast cancer; mPFS: median progression-free survival; m: months; AEs: adverse events; mOS: median overall survival; ASAT: aspartate aminotransferase; T-DXd: trastuzumab deruxtecan; ILD: interstitial lung disease; T: trastuzumab; ORR: overall response rate; CR: complete remission; CT: chemotherapy; ICIs: immune checkpoint inhibitors; mUC: metastatic urothelial carcinoma; EV: enfortumab vedotin; SG: sacituzumab govitecan; HR: hormonal receptor; ET: endocrine therapy; TNBC: triple-negative breast cancer; NSCLC: non-small-cell lung cancer; pts: patients; nb: number.

**Table 2:** Mechanisms of resistance and of the overcoming of resistance.

Mechanism of Resistance		Overcoming Resistance
Antigen-related resistance	■ Reduced HER2/NECTIN 4 levels ■ Tumor heterogeneity ■ Truncated forms of antigen ectodomain ■ Parallel genomic alteration ■ Heregulin	■ Bystander effect ■ Biparatropic ADC
Payload-related resistance	■ DAR ■ Payload conjugation	■ Payload diversification: replacement of payload (auristatin vs. anthracycline based, topoisomerase I vs. belotecan derivative) ■ Lysine vs. cysteine conjugate
Altered internalization	■ Microenvironment and enzymatic role	
Impaired lysosomal function	■ Lysosomal pH	■ Replacement of hydrophobic with hydrophilic linkers
Overexpression of drug-efflux pumps	■ ATP-binding cassette (ABC) transporters	■ Aberrant expression of transporters ■ Transporter inhibition
Activation of signaling pathways	■ PI3K/AKT signaling activation ■ Wnt/β-catenin pathway	■ Combination therapies

**Table 3:** Ongoing trials evaluating T-DXd in combination with ICIs and TKIs.

Study	Drugs	Phase	No. of pts.	Population	Primary Endpoints
NCT05480384 (BrUOG 413) [102]	T-DXd + nivolumab	II	25	Adjuvant treatment after trimodality treatment in HER2+ esophagus and GEJ	Safety
NCT04539938 (HER2CLIMB-04) [103]	T-DXd + tucatinib	II	70	HER2+ MBC after progression on taxane + T	ORR
NCT05633979 [104]	T-DXd + valemetostat	Ib	37	Low-HER2 /ultra-low/null MBC	Safety, MTD, ORR, RDE
NCT05795101 (TRUDI) [105]	T-DXd + durvalumab	II	63	First-line HER2+/low inflammatory breast cancer	pCR
NCT05372614 [106]	T-DXd + neratinib	I	18	Metastatic HER2-altered cancers	DLTs TEAEs
NCT04704661 (DASH) [107]	T-DXd + AZD6738	I	15	Advanced solid tumors with HER2 expression	Safety, RP2D
NCT04538742 (DB-07) [108]	T-DXd + durvalumab or pertuzumab or paclitaxel or tucatinib	I/II	245	First-line HER2+ advanced and/or MBC	AEs, SAEs
NCT04042701 [109]	T-DXd + pembrolizumab	I	115	MBC or NSCLC	DLTs, ORR
NCT04686305 (DESTINY-Lung03) [110]	T-DXd + durvalumab + pemetrexed or platinum	Ib	136	First-line HER2+ advanced or metastatic NSCLC	AEs SAEs
NCT04379596 (DESTINY-Gastric03) [111]	T-DXd alone or + durvalumab or pembro and CT	Ib/II	351	HER2+ previously treated or untreated gastric and GEJ or esophageal cancer patients	AEs, SAEs, ORR
NCT04585958 [112]	T-DXd + olaparib	I	55	Metastatic HER2-expressing cancers	MTD, RP2D, AEs
NCT03334617 (HUDSON) [113]	Cohort T-DXd + durvalumab	II	570	NSCLC progressed on anti-PD-1/PD-L1 containing therapy	ORR

RDE: recommended dose for expansion; T-DXd: trastuzumab deruxtecan; MBC: metastatic breast cancer; NSCLC: non-small-cell lung cancer; DLTs: dose-limiting toxicities; ORR: overall response rate; AEs: adverse events; SAEs: serious adverse events; MTD: maximum tolerated dose; RP2D: recommended phase 2 dose; GEJ: gastro-esophageal junction; PD-1: programmed death-1; PD-L1: programmed death ligand-1; TEAEs: treatment-emergent adverse events; pCR: pathologic complete response; CT: chemotherapy; pts: patients; nb: number.

**Table 4:** Ongoing trials of T-DM1 and SG in combination with ICIs and TKIs.

Study	Drugs	Phase	No. of pts.	Population	Primary Endpoints
NCT05673928 (TUCATEMEB) [114]	T-DM1 + tucatinib	II	30	HER2+ metastatic solid tumors and brain metastases	Intracranial antitumor activity
NCT03975647 (HER2CLIMB-02) [115]	T-DM1 + tucatinib or placebo	III	565	Metastatic HER2+ BC with history of prior taxane and T	PFS
NCT04873362 (Astefania) [116]	T-DM1 + atezolizumab or placebo	III	1700	Adjuvant treatment for HER2+ BC with high risk of recurrence	IDFS
NCT04457596 (CompassHER2 RD) [117]	T-DM1 + tucatinib or placebo	III	1031	HER2+ BC with residual disease after neoadjuvant HER2-directed therapy	IDFS
NCT05560308 [118]	T-DM1 + pyrotinib maleate	II	50	HER2+ MBC progressed on TKI therapy	ORR
NCT05143229 (ASSET) [119]	SG + aleplisib	I	18	Metastatic HER2- BC	RP2D
NCT04724018 (DAD) [120]	SG + EV	I	24	Metastatic UC progressiong on platinum-based CT and PD-1/PD-L1 inhibitors	MTD, DLTs
NCT04434040 (ASPRIA) [121]	SG + atezolizumab	II	40	Residual invasive disease in TNBC following neoadjuvant CT	Undetectable ctDNA
NCT04468061 (Saci-IO TNBC) [122]	SG with or without pembrolizumab	II	110	First-line PD-L1 negative metastatic TNBC	PFS
NCT04448886 (Saci-IO HR+) [123]	SG with or withour pembrolizumab	II	110	Metastatic HR+/HER2- BC	PFS
NCT04039230 [124]	SG + talazoparib	I/II	75	Metastatic TNBC	DLT
NCT04826341 [125]	SG + berzosertib	I/II	85	SCLC and HRD cancers resistant to PARP inhibitors	ORR, MTD
NCT05609968 (MK-3475-D46) [126]	SG + pembrolizumab vs. pembrolizumab	III	614	First-line metastatic NSCLC with PD-L1 TPS ≥ 50%	PFS, OS
NCT04863885 [127]	SG + nivo + ipi	I/II	46	First line for cisplatin-ineligible mUC	MTD, ORR
NCT05633654 (ASCENT-05) [128]	SG + pembrolizumab vs. capecitabine	III	1514	TNBC with residual invasive disease after surgery and neoadjuvant therapy	IDFS
NCT03547973 (TROPHY U-01) [129]	SG alone or + pembrolizumab or cisplatin + avelumab or cisplatin + zimberelimab	II	643	Unresectable mUC	ORR, PFS
NCT05535218 (SURE-02) [130]	SG + pembrolizumab	II	48	High-risk localized bladder cancer	cPR
NCT05382286 (ASCENT-04) [131]	SG + pembrolizumab vs. TPO	III	440	First-line metastatic TNBC whose tumors express PD-L1	PFS
NCT05186974 (EVOKE-02) [126]	SG + pembro + platinum-based	II	224	First-line metastatic NSCLC without genomic alterations	ORR, DLTs
NCT03971409 (InCITe) [132]	SG + immunotherapy	II	150	Metastatic TNBC	ORR

IDFS: invasive disease-free survival; CPR: complete pathological response; TPO: treatment of physician’s choice; T-DM1: trastuzumab emtansine; BC: breast cancer; PFS: progression-free survival; cPR: complete pathological response; ORR: overall response rate; DLTs: dose-limiting toxicities; TNBC: triple-negative breast cancer; NSCLC: non-small-cell lung cancer; SG: sacituzumab govitecan; PD-L1: programmed death ligand-1; mUC: metastatic urothelial carcinoma; MTD: maximum tolerated dose; OS: overall survival; TPS: tumor-proportion score; SCLC: small-cell lung cancer; CT: chemotherapy; ctDNA: circulating tumor DNA; T: trastuzumab; EV: enfortumab vedotin; TKI: tyrosine-kinase inhibitor; PD-1: programmed death-1; HRD: homologous recombinant deficiency; PARP: poly-ADP ribose polymerase.

**Table 5:** Ongoing trials of EV in combination with ICIs and TKIs.

Study	Drugs	Phase	No. of pts.	Population	Primary Endpoints
NCT05239624 (EV-ECLIPSE) [133]	EV + pembrolizumab	II	23	LA- or node-positive UC before surgery	pCR rate
NCT04963153 [134]	EV + erdafitinib	Ib	30	Metastatic UC progressed on platinul and PD1/L1 inhibitors with FGFR2/3 alterations	AEs; RP2D; MTD
NCT05524545 (ASPEN-07) [135]	EV + evorpacept (ALX148)	I	30	Mettastatic UC progressed on platinul and PD1/L1 inhibitors	DLTs; AEs
NCT05775471 [136]	Pembrolizumab + EV followed by pembrolizumab	II	21	Neoadjuvant before radical nephroureterectomy for high-risk UTUC followed by adjuvant pembrolizumab	ORR; RFS
NCT03924895 (KEYNOTE-905/EV-303) [137]	Pembrolizumab alone or pembrolizumab + EV or nothing	III	857	Perioperative treatment before cystectomy in cisplatin-ineligible or cisplatin-declining patients with MIBC	EFS
NCT04878029 [138]	EV +cabozantinib	Ib	32	Metastatic UC after progression on platine and ICIs	RP2D
NCT04960709 (VOLGA) [139]	Durva + treme + EV vs. durva + EV	III	830	MIBC ineligible for cisplatin or who refused cisplatin	pCR rate; EFS; AEs
NCT04223856 (EV-302) [140]	EV + pembrolizumab vs. chemotherapy	III	990	Previously untreated LA or metastatic UC	PFS; OS
NCT04700124 (KEYNOTE-B15/EV-304) [141]	Perioperative EV + pembrolizumab vs. neoadjuvant chemotherapy	III	784	Cisplatin-eligible MIBC	EFS

EV: enfortumab vedotin; LA: locally advanced; UC: urothelial carcinoma; pCR: pathologic complete response; AEs: adverse events; RP2D: recommended phase 2 dose; MTD: maximum tolerated dose; PD-1: programmed death-1; PD-L1: programmed death ligand-1; DLTs: dose-limiting toxicities; UTUC: upper-tract urothelial carcinoma; ORR: overall response rate; RFS: relapse-free survival; MIBC: muscle-invasive bladder cancer; EFS: event-free survival; ICIs: immune-checkpoint inhibitors; PFS: progression-free survival; OS: overall survival.
